# Genome Sequence of Phytophthora fragariae var. fragariae, a Quarantine Plant-Pathogenic Fungus

**DOI:** 10.1128/genomeA.00034-15

**Published:** 2015-03-26

**Authors:** Ruifang Gao, Yinghui Cheng, Ying Wang, Ying Wang, Liyun Guo, Guiming Zhang

**Affiliations:** a Animal and Plant Inspection and Quarantine Technology Center, Shenzhen Entry-Exit Inspection and Quarantine Bureau, Shenzhen, China; b Shenzhen Key Laboratory for Research and Development on Detection Technology of Alien Pests, Shenzhen Academy of Inspection and Quarantine, Shenzhen, China; c College of Agronomy and Biotechnology, China Agricultural University, Beijing, China

## Abstract

*Phytophthora* fragariae var. fragariae is a serious plant-pathogenic fungus causing red core disease in strawberries, resulting in a larger number of fruit produced, and the fungus has been regulated as a quarantine pest of many countries and regions. Here, we announce the genome sequence of P. fragariae var. fragariae, and this information might provide insight into the mechanism of pathogenicity and host specificity of this pathogen, as well as help us further identify targets for fungicides.

## GENOME ANNOUNCEMENT

*Phytophthora* contains >100 species, all plant pathogens, which are the most important plant-pathogenic fungi (1, 2). The pathogen Phytophthora fragariae Hickman is the causal agent of red core disease in strawberries. Its hosts contain all subspecies of P. fragariae and perhaps certain species of Rubus, such as loganberries and blackberries (Rubus fruticosus) (3). The symptoms of the disease can include dwarfism, wilting of leaves and petioles, reddening of the root stele, and death of the plant. The infective agent is the motile zoospore, which is attracted to host material. In general, the fungus lacks any aerial dispersal mechanism, but it has the ability to spread locally in drainage or irrigation water and to persist as long-lived oospores in soil or in planting material. It can wipe out entire plantations in only a few years, effectively ruining a starting raspberry farm, so it is a major factor limiting fruit production (4). It was officially regulated as a quarantine pest of many countries and regions, such as the European Union (EU), European and Mediterranean Plant Protection Organization (EPPO) (A2 list), the United States, and China.

The fungal strain used in this study, P. fragariae var. fragariae 309.62, was obtained from the Centraalbureau voor Schimmelcultures (CBS). Whole-genome sequencing was performed using a genome strategy employing shotgun and paired-end genome libraries. The shotgun library was sequenced using the Ion Torrent Personal Genome Machine (PGM) (Life Technologies) and the paired-end library using the Roche/Illumina HiSeq 2500 sequencer. We obtained 18,269,515 shotgun and 18,902,113 paired-end reads, resulting in approximately 105-fold coverage. They were assembled into 1,616 scaffolds by Newbler Assembler 2.9 (454 Life Sciences, Branford, CT). The total size of the assembled genome of P. fragariae is 73.68 Mb, with a G+C content of 53.25%.

A total of 18,692 protein-coding genes were predicted based on the homologies to known genes by applying GLEAN (5). Gene annotation was predicted and comparative analysis performed by applying a combination of BLAST (6) against Swiss-Prot (*E* values, ≤ 1e - 10), COG (*E* values, ≤ 1e - 10), and KEGG (*E* values, ≤ 1e - 10). InterPro domains were annotated by InterProScan 4.8 (7, 8), and functional assignments were classified using Gene Ontology (9).

The genome sequence of P. fragariae is a valuable resource for identifying the molecular mechanism of pathogenicity, evolutionary status, and host specificity of the species by facilitating the isolation of novel virulence and avirulence genes, as well as helping identify targets for fungicides. These valuable bioinformatic data will also serve as platform to facilitate comparative genomic studies involving plant-pathogenic fungi. The research article about this plant-pathogenic fungus will be published in the future.

### Nucleotide sequence accession number.

This whole-genome project has been deposited in GenBank under the accession no. JHVZ00000000. The version described in this paper is the third version.
